# Supplementary material for: Downregulated RBM5 Enhances CARM1 Expression and Activates the PRKACA/GSK3β Signaling Pathway through Alternative Splicing-Coupled Nonsense-Mediated Decay
Source: Cancers (Basel). 2023 Dec 27;16(1):139. doi: 10.3390/cancers16010139 (PMC10778212; doi:10.3390/cancers16010139)
Supplement: Supplementary file 1 [file cancers-16-00139-s001.zip › CV of all authors.pdf]

CV of all authors

Yan-ping Zhang, Ph.D, Department of Urology, the Second Hospital of Hebei Medical University, Shijiazhuang, Hebei, 050011, PR China.

Email: zhangyanping@hebmu.edu.cn. Tel: +86-0311-66002996

Fang Li, Ph.D, Third Division, Department of Cardiology, The Second Hospital of Hebei Medical University, Shijiazhuang, Hebei, 050011, PR China.

Email: lifangfei3320@yeah.net. Tel: +86-0311-66003981

Zhen-wei Han, Ph.D, Department of Urology, the Second Hospital of Hebei Medical University, Shijiazhuang, Hebei, 050011, PR China.

Email: hanzhenwei@hebmu.edu.cn. Tel: +86-0311-66002996

Zhi-hai Teng, M.D, Department of Urology, the Second Hospital of Hebei Medical University, Shijiazhuang, Hebei, 050011, PR China.

Email: tengzhihai1988@126.com. Tel: +86-0311-66002996

Cheng-gen Jin, Ph.D, Department of Urology, the Second Hospital of Hebei Medical University, Shijiazhuang, Hebei, 050011, PR China.

Email: 29005698@hebmu.edu.cn. Tel: +86-0311-66002996

Hao Yuan, B.S, Department of Urology, the Second Hospital of Hebei Medical University, Shijiazhuang, Hebei, 050011, PR China.

Email: 1490882758@qq.com. Tel: +86-0311-66002996

Si-hao Zhang, B.S, Department of Urology, the Second Hospital of Hebei Medical University, Shijiazhuang, Hebei, 050011, PR China.

Email: 3223140950@qq.com. Tel: +86-0311-66002996

Ke-xin Sun, B.S, Department of Urology, the Second Hospital of Hebei Medical University, Shijiazhuang, Hebei, 050011, PR China.

Email: 506031387@qq.com. Tel: +86-0311-66002996

Ya-xuan Wang, Ph.D, Department of Urology, the Second Hospital of Hebei Medical University, Shijiazhuang, Hebei, 050011, PR China.

Email: 27000413@hebmu.edu.cn. Tel: +86-0311-66002996

河北医科大学第二医院 科研处

河北医科大学第二医院 1301051101209

THE SECOND HOSPITAL OF HEBEI MEDICAL UNIVERSITY * HEBEI CHINA *
