# Supplementary material for: Downregulated RBM5 Enhances CARM1 Expression and Activates the PRKACA/GSK3β Signaling Pathway through Alternative Splicing-Coupled Nonsense-Mediated Decay
Source: Cancers (Basel). 2023 Dec 27;16(1):139. doi: 10.3390/cancers16010139 (PMC10778212; doi:10.3390/cancers16010139)
Supplement: Supplementary file 1 [file cancers-16-00139-s001.zip › MDPI-author-signatures.pdf]

Author statements

Please insert the relevant text under the subheadings below. A completed form must be signed by all authors. Please note that we only accept hand-signed signatures.

Manuscript title: Downregulated RBM5 Enhances CARM1 Expression and Activates PRKACA/GSK3β Signaling Pathway Through Alternative Splicing-Coupled Nonsense-Mediated Decay

Authors: Yan-ping Zhang, Fang Li , Zhen-wei Han , Zhi-hai Teng , Cheng-gen Jin , Hao Yuan , Si-hao Zhang , Ke-xin Sun and Ya-xuan Wang

Corresponding author: Ya-xuan Wang

Article type: Article

If your manuscript contains information related to human or animal use, please clearly state:

1. Does your study need ethic approval?

   Yes ☑

   No ☐ If no, please state the reason:

2. If ethic approval is needed, do you have an ethic approval number?

   Yes ☑ , please enter ethic approval number here: 2020-R318

   No ☐ If no, please provide scanned copy of ethic approval files

Authors' contributions

We follow International Committee of Medical Journal Editors (ICMJE) recommendation of authorship, which is based on the following 4 criteria:

1. Substantial contributions to the conception or design of the work; or the acquisition, analysis, or interpretation of data for the work; AND
2. Drafting the work or revising it critically for important intellectual content; AND
3. Final approval of the version to be published; AND
4. Agreement to be accountable for all aspects of the work in ensuring that questions related to the accuracy or integrity of any part of the work are appropriately investigated and resolved.

All those designated as authors should meet all four criteria for authorship, and all who meet the four criteria should be identified as authors. Those who do not meet all four criteria should be acknowledged. Please insert here the contribution each author made to the manuscript—e.g., literature search, figures, study design, data collection, data analysis, data interpretation, writing etc. If all authors contributed equally, please state this. The information provided here must match the contributors' statement in the manuscript.

MDPI

Author Contributions:

Yan-ping Zhang carry out the design and conception, the methodology, acquisition of the data, analysis and interpretation of the data, writing, review and revision of the manuscript.

Fang Li carry out the design and conception, the methodology, and acquisition of the data.

Zhen-wei Han carry out the methodology, analysis and interpretation of the data.

Zhi-hai Teng carry out the methodology and animal models.

Cheng-gen Jin carry out acquisition of the data.

Hao Yuan carry out acquisition of the data.

Si-hao Zhang carry out acquisition of the data and animal models.

Ke-xin Sun carry out animal models.

Conflicts of interest

Please insert here: 1. authors' conflicts of interest; 2. sources of support for the work, including sponsor names along with explanations of the role of those sources if any in study design; collection, analysis, and interpretation of data; writing of the report; 3. the decision to submit the report for publication; or a statement declaring that the supporting source had no such involvement, etc.

This study was supported by the Natural Science Foundation of Hebei Provence(Grant H2021206080), The Health Commission of Hebei Provence(20180269,20200937)

The authors declare no conflicts of interest.

I agree with: the plan to submit/publish to *Cancers;* the contents of the manuscript; to being listed as an author; and to the conflicts of interest statement as summarised. I have had access to all the data in the study (for original research articles) and accept responsibility for its validity.

Title and name: Yan-ping zhang Highest degree: Signature: Yan-ping Zhang Date: Dec. 11. 2023

Title and name: Fang Li Highest degree: Signature: Fang Li Date: Dec. 11. 2023

Title and name: Zhen-wei Han Highest degree: Signature: zhen-wei Han Date: Dec. 11. 2023

Title and name: Zhi-hai Teng Highest degree: Signature: Zhi-hai Teng Date: Dec. 11. 2023

Title and name: Cheng-gen Jin Highest degree: Signature: Cheng-gen Jin Date: Dec. 11. 2023

Title and name: Hao Yuan Highest degree: Signature: Hao Yuan Date: Dec. 11. 2023

Title and name: Si-hao Zhang Highest degree: Signature: si hao — zhang Date: Dec. 11. 2023

Title and name: ke-xin Sun Highest degree: Signature: ke-xin Sun Date: Dec. 11. 2023

Title and name: Ya-xuan Wang Highest degree: Signature: Ya-xuan Wang Date: Dec. 11. 2023

Title and name: Highest degree: Signature: .... Date:

Corresponding author declaration

I Yaxuan Wang, the corresponding author of this manuscript, certify that the contributors' and conflicts of interest statements included in this paper are correct and have been approved by all co-authors.
