# Supplementary material for: Downregulated RBM5 Enhances CARM1 Expression and Activates the PRKACA/GSK3β Signaling Pathway through Alternative Splicing-Coupled Nonsense-Mediated Decay
Source: Cancers (Basel). 2023 Dec 27;16(1):139. doi: 10.3390/cancers16010139 (PMC10778212; doi:10.3390/cancers16010139)
Supplement: Supplementary file 1 [file cancers-16-00139-s001.zip › Original Data of Western blot FigS2-S18.pdf]

Supplementary materials

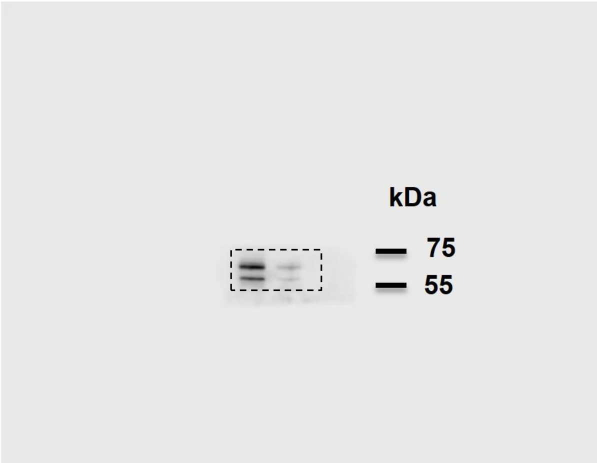

CARM1

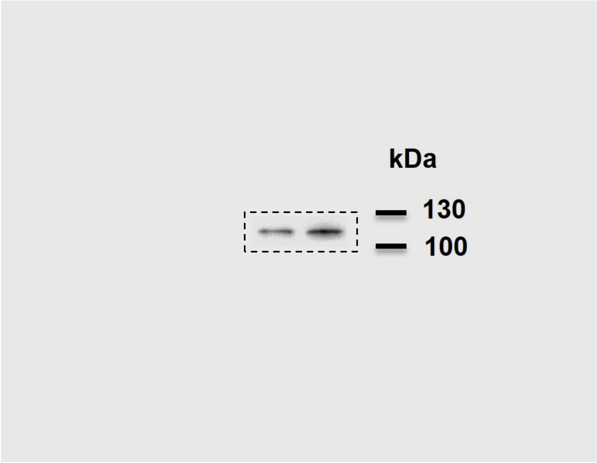

RBM5

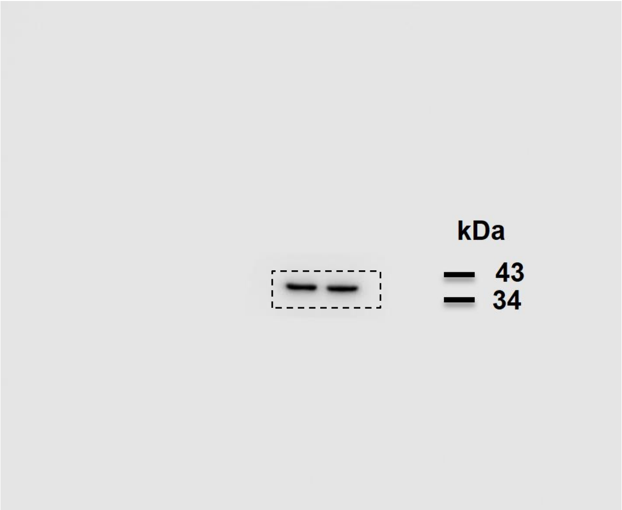

β-actin

Supplementary Figure S2. The original immunoblots of Figure 1D.

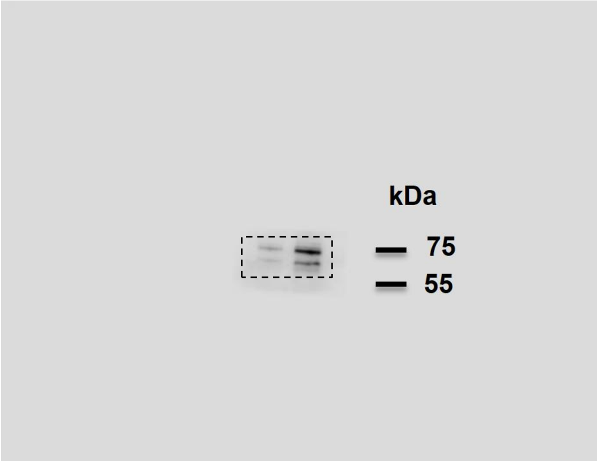

CARM1

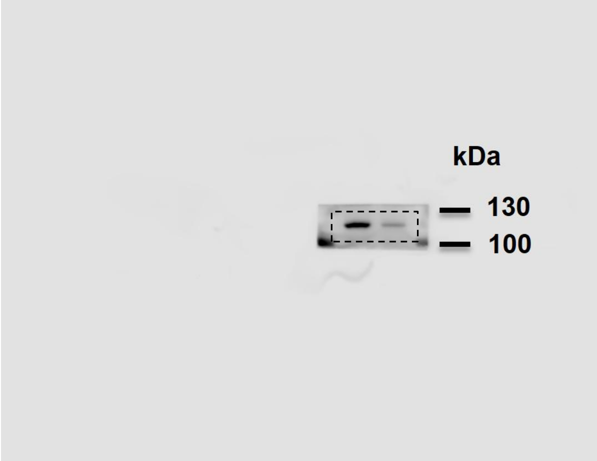

RBM5

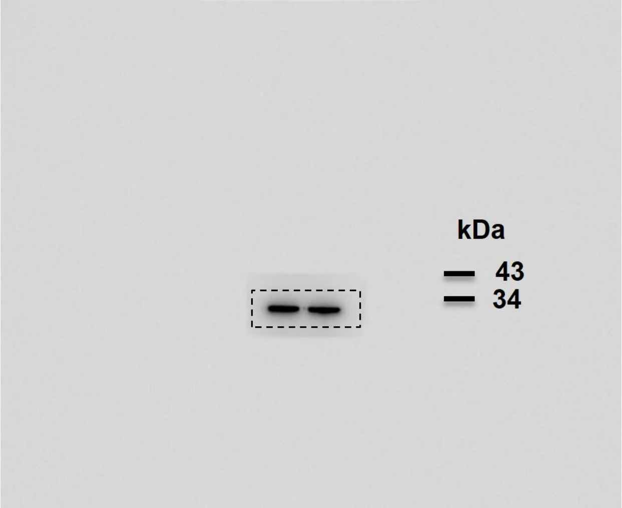

β-actin

Supplementary Figure S3. The original immunoblots of Figure 1F.

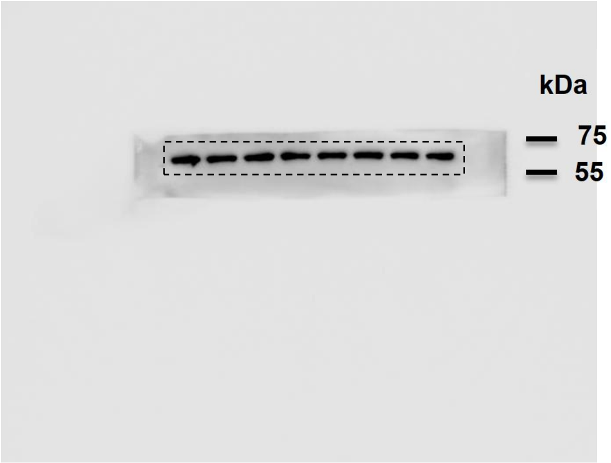

CARM1

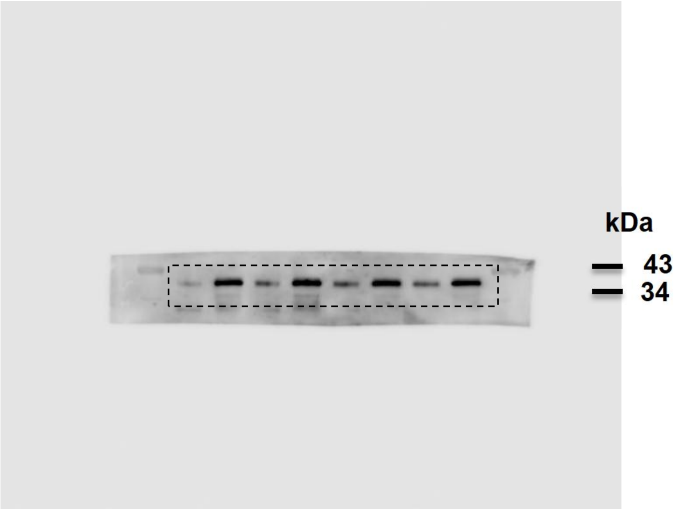

β-actin

Supplementary Figure S4. The original immunoblots of Figure 1J.

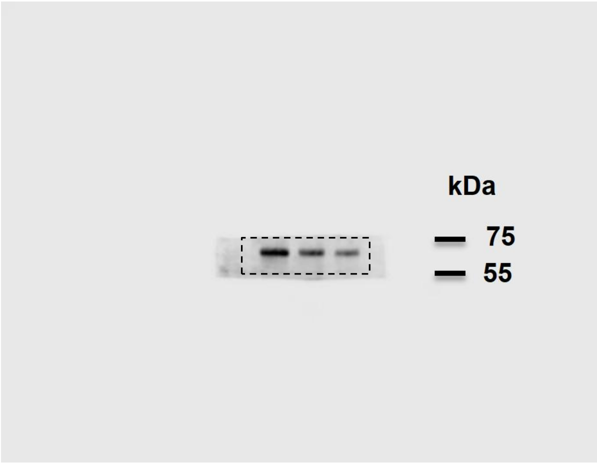

CARM1

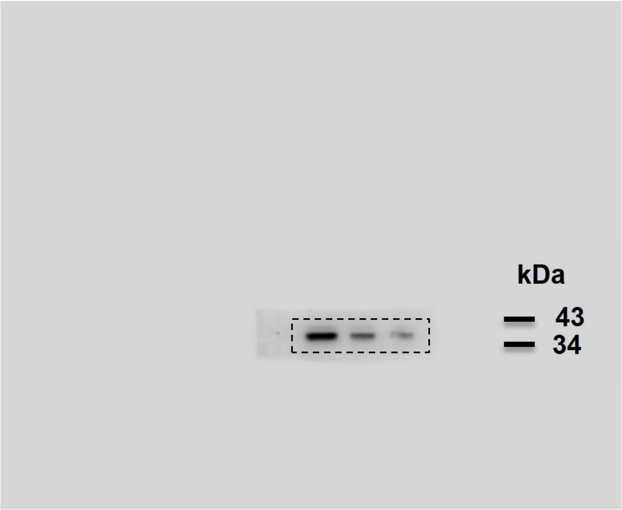

CDK4

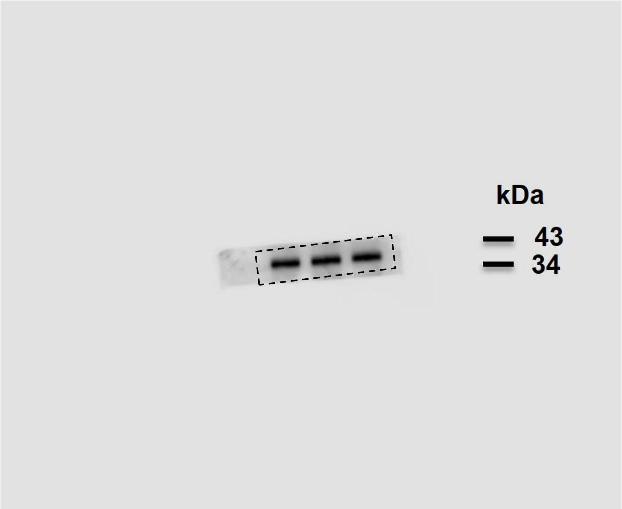

β-actin

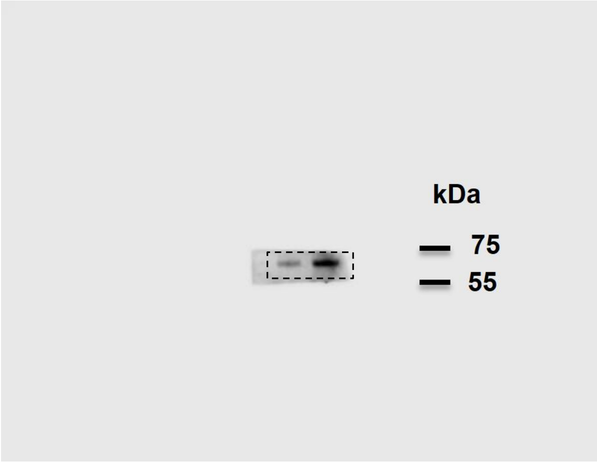

CARM1

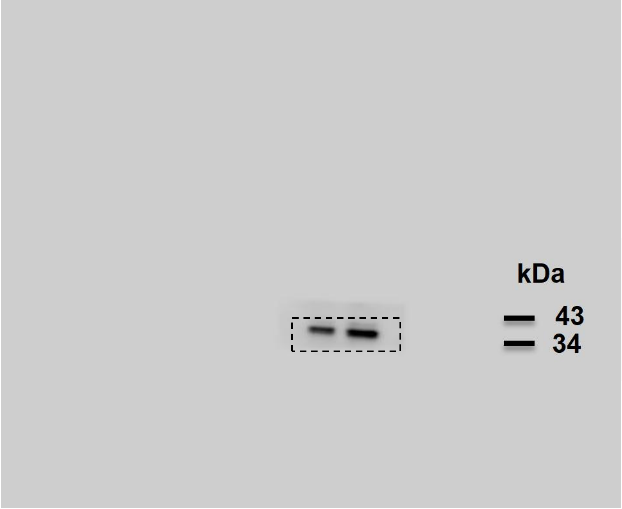

CDK4

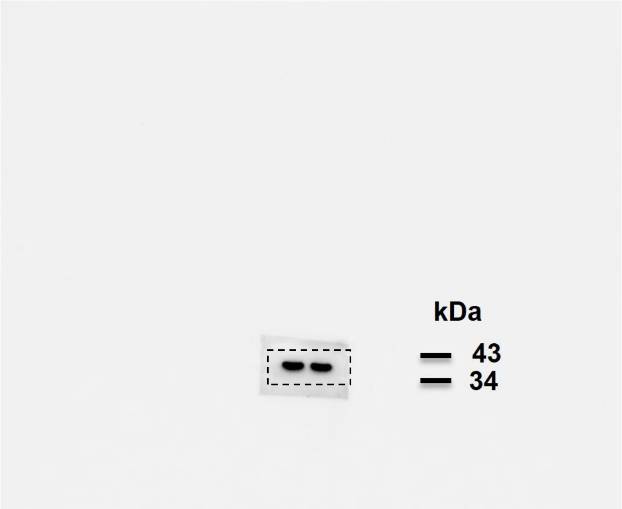

β-actin

Supplementary Figure S5. The original immunoblots of Figure 2A.

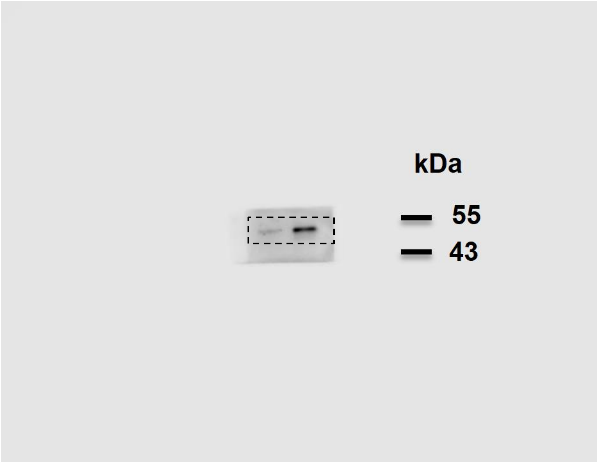

pGSK3β

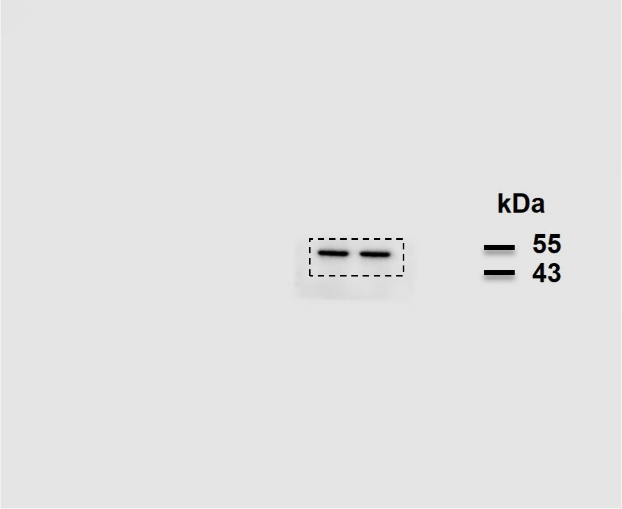

GSK3β

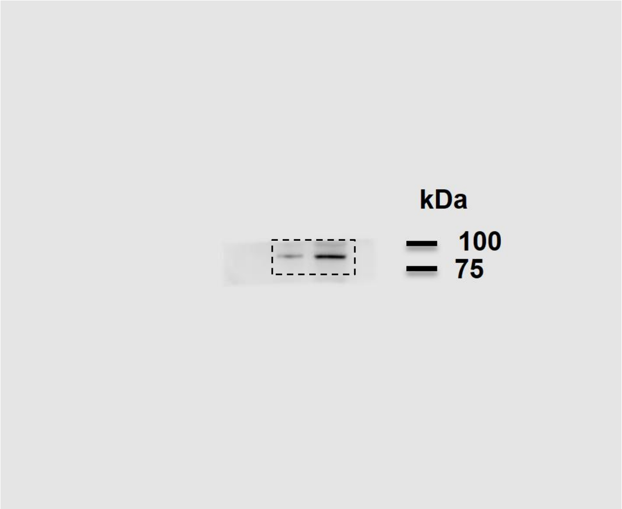

β-catenin

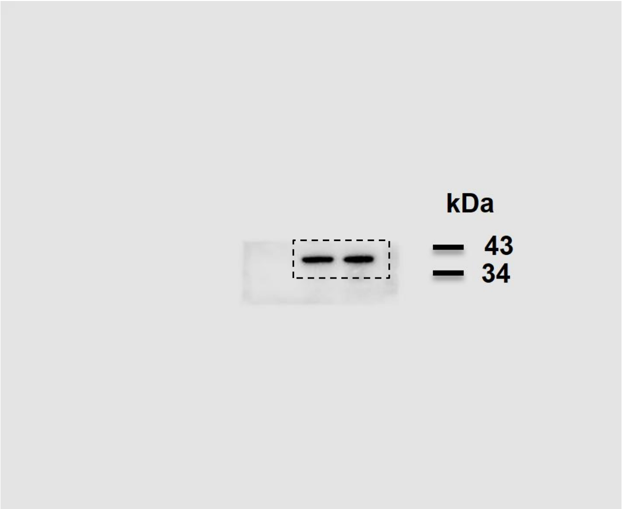

β-actin

Supplementary Figure S6. The original immunoblots of Figure 3A.

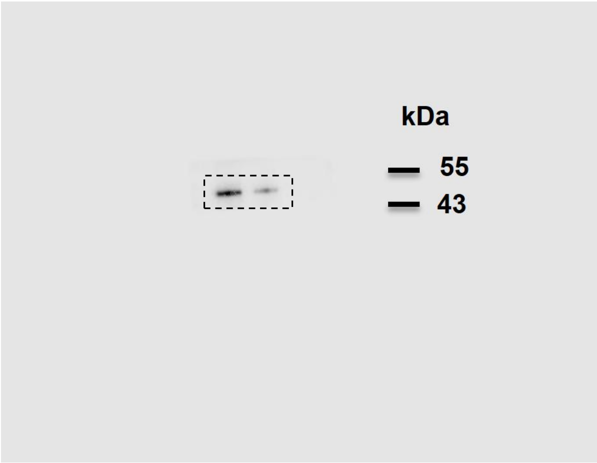

pGSK3β

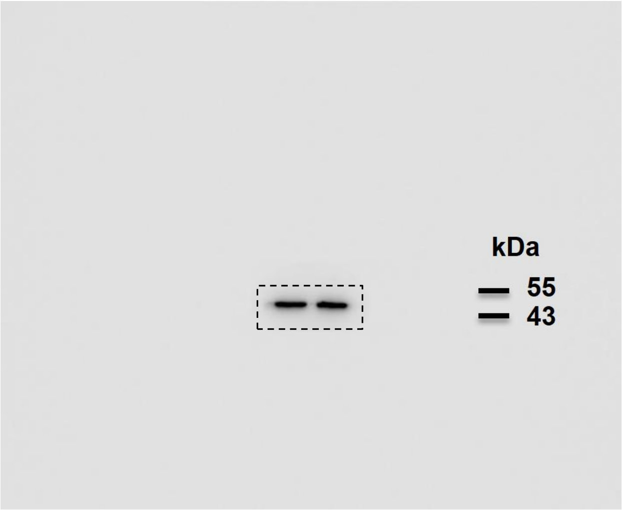

GSK3β

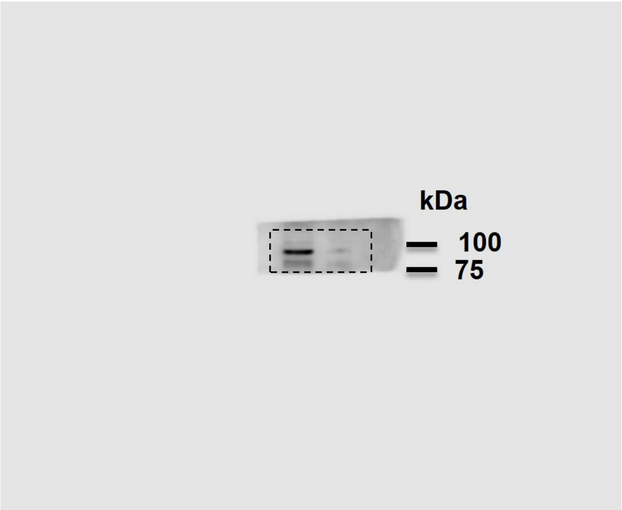

β-catenin

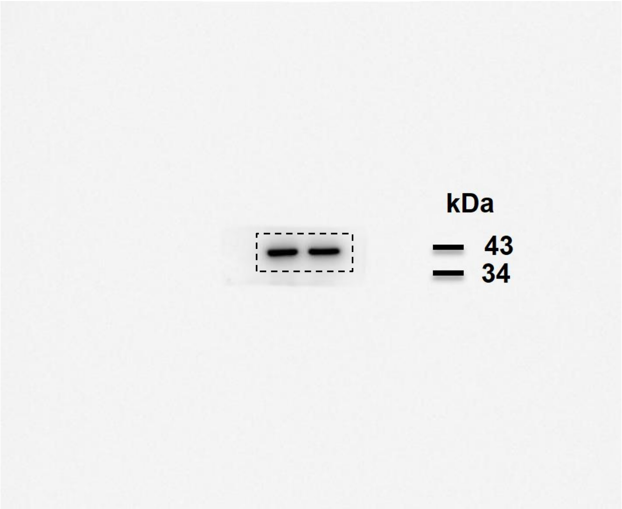

β-actin

Supplementary Figure S7. The original immunoblots of Figure 3C.

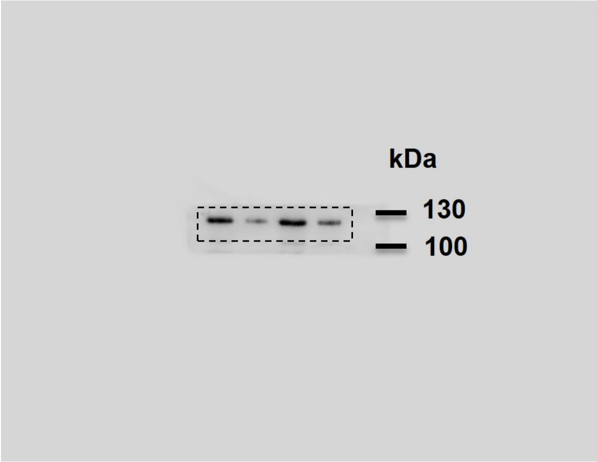

RBM5

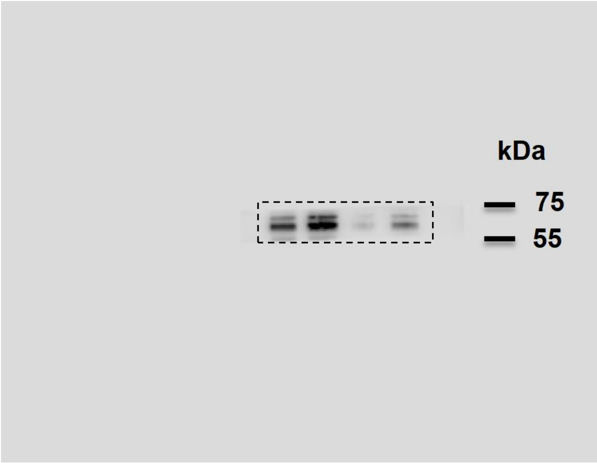

CARM1

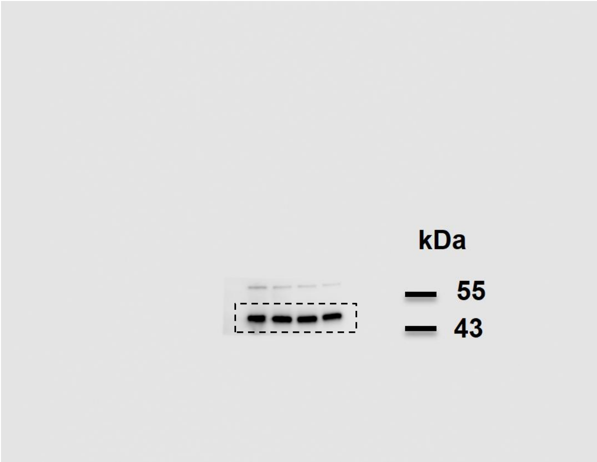

pGSK3β

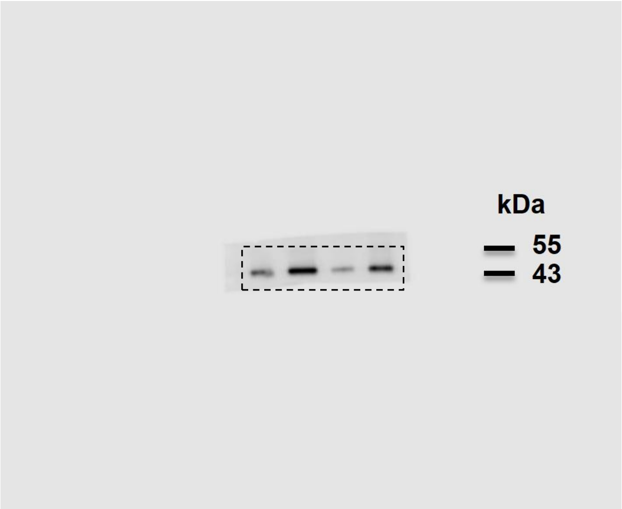

GSK3β

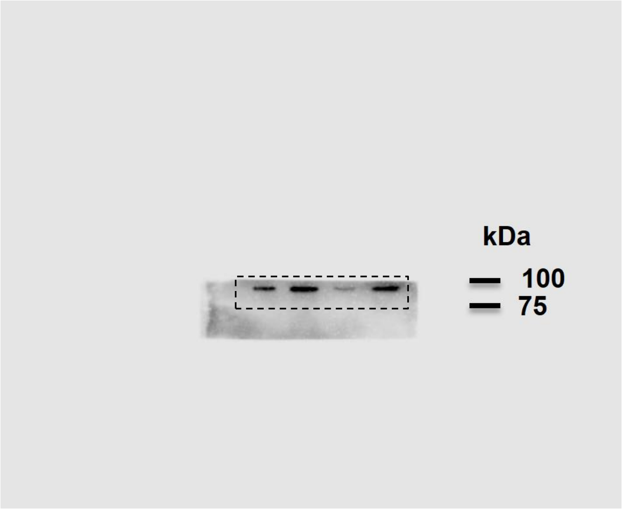

β-catenin

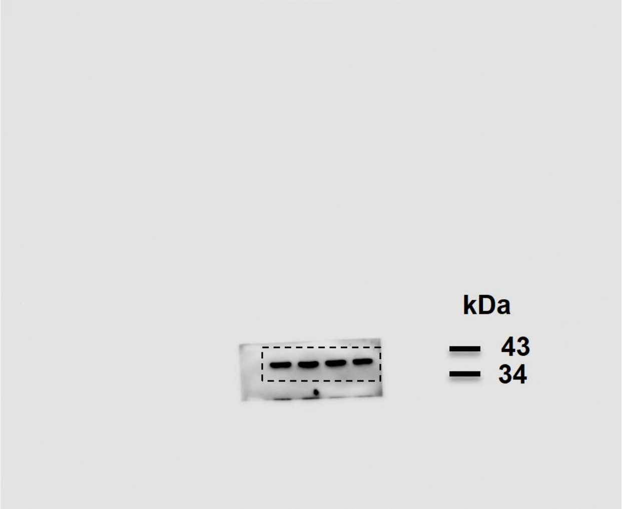

β-actin

Supplementary Figure S8. The original immunoblots of Figure 3E.

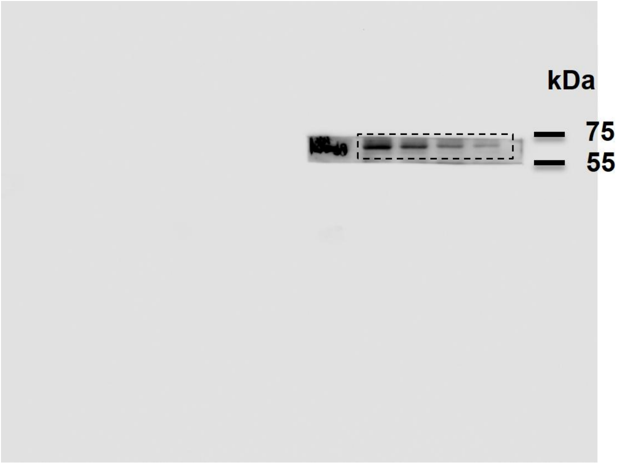

CARM1

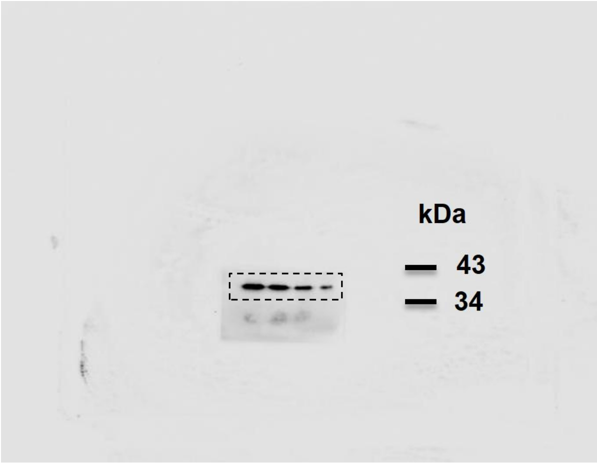

CDK4

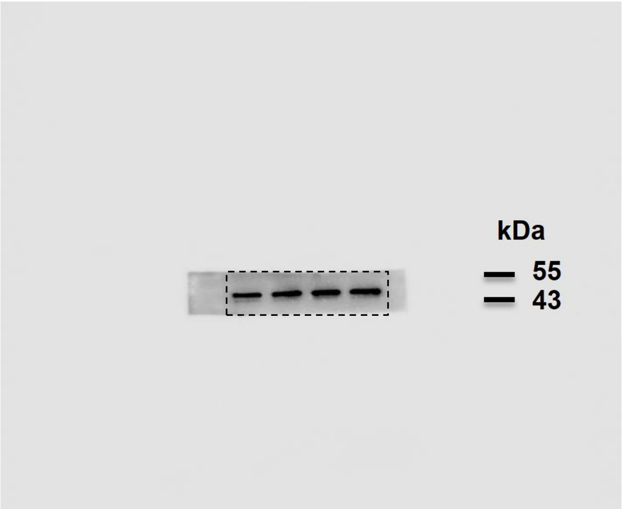

GSK3β

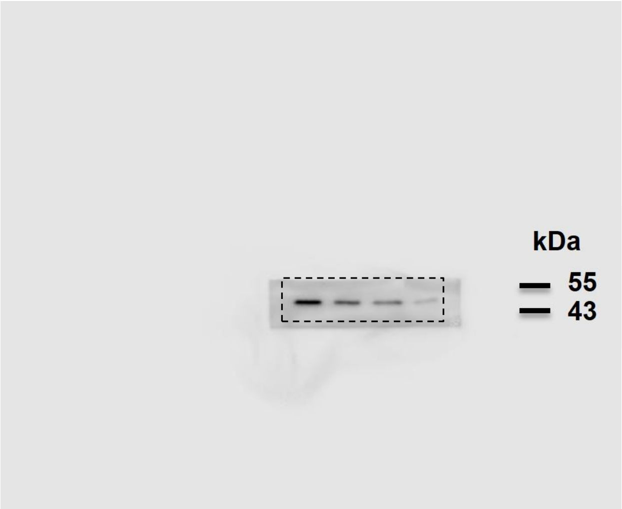

pGSK3β

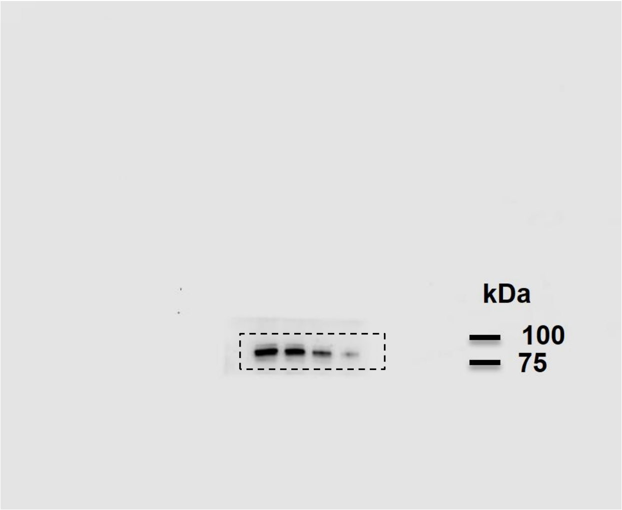

β-catenin

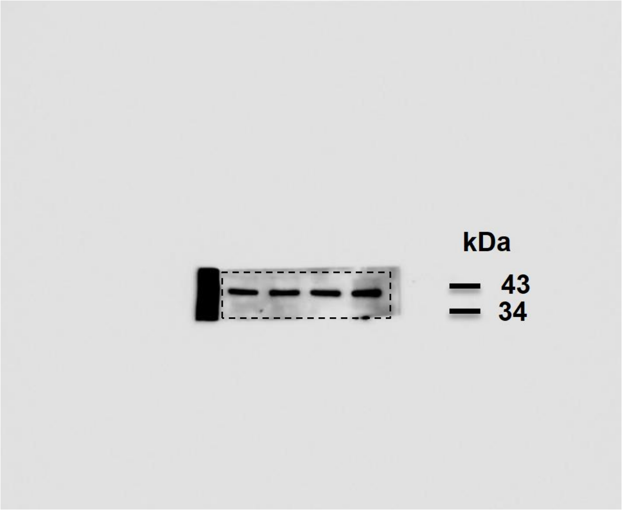

β-actin

Supplementary Figure S9. The original immunoblots of Figure 3G.

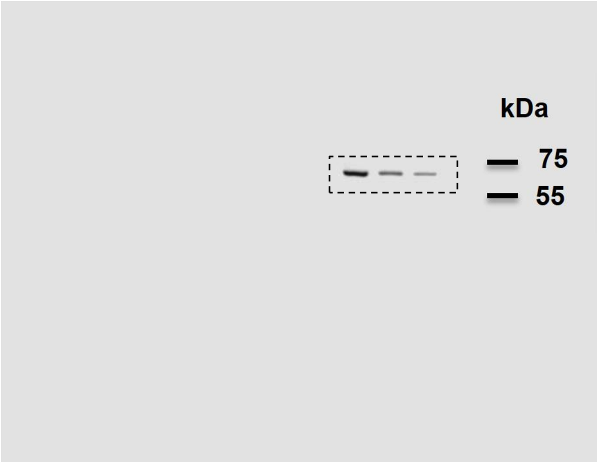

CARM1

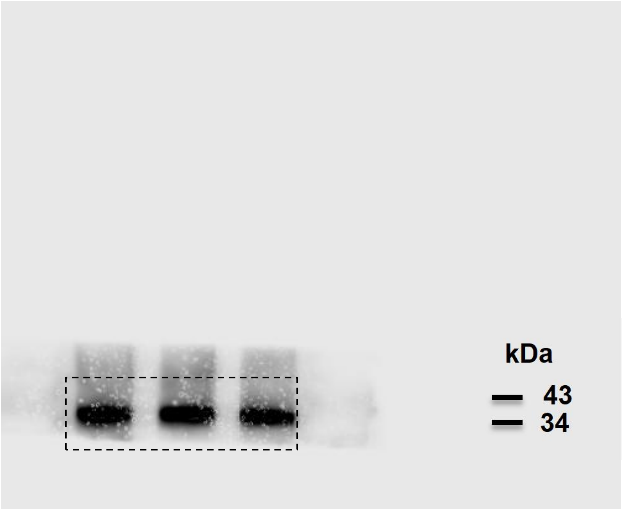

β-actin

Supplementary Figure S10. The original immunoblots of Figure 4E.

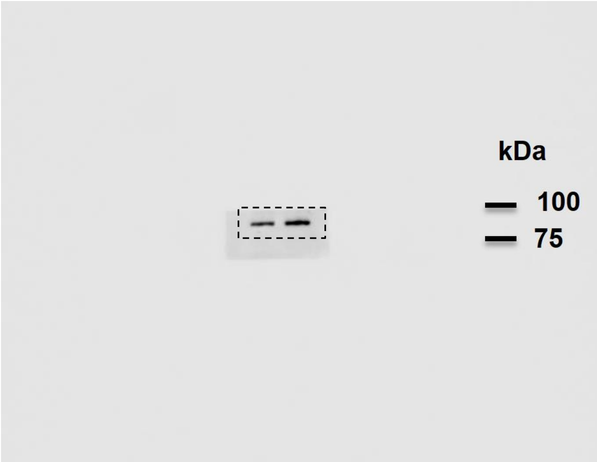

PRKACA

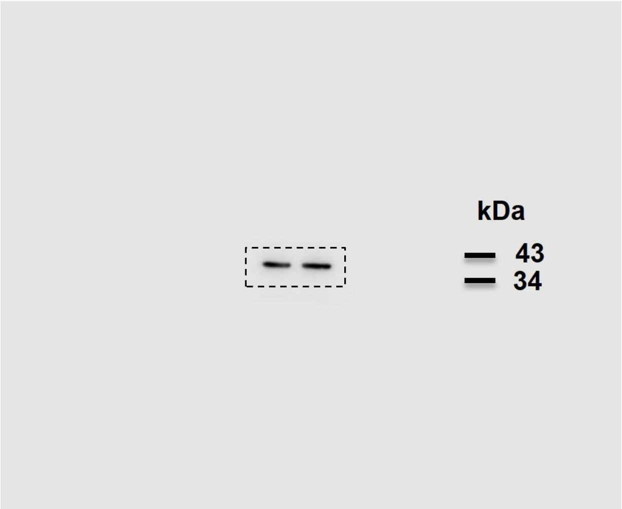

β-actin

Supplementary Figure S11. The original immunoblots of Figure 5C.

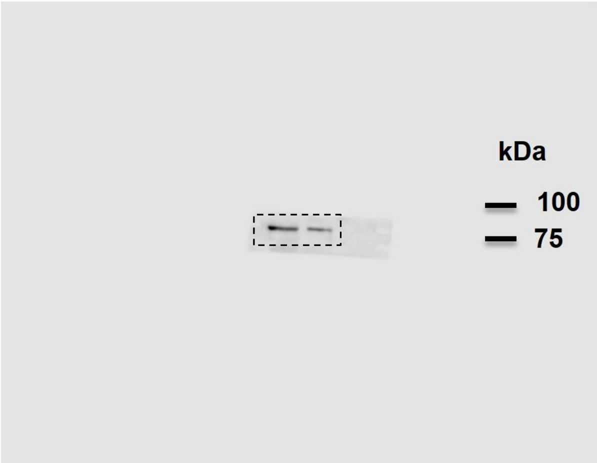

PRKACA

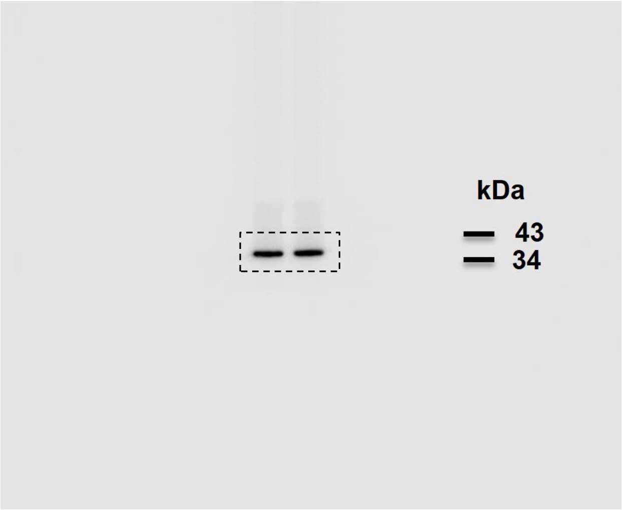

β-actin

Supplementary Figure S12. The original immunoblots of Figure 5E.

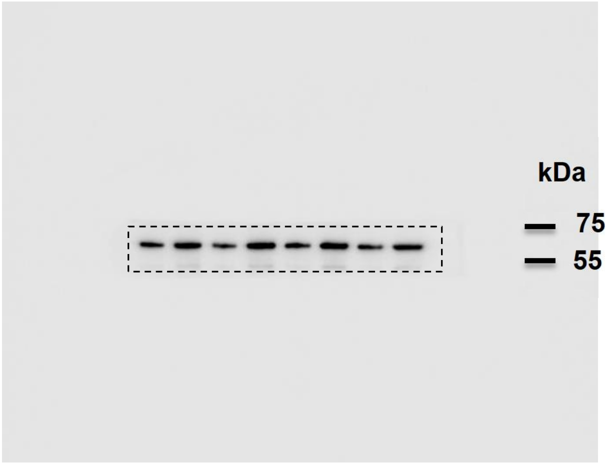

PRKACA

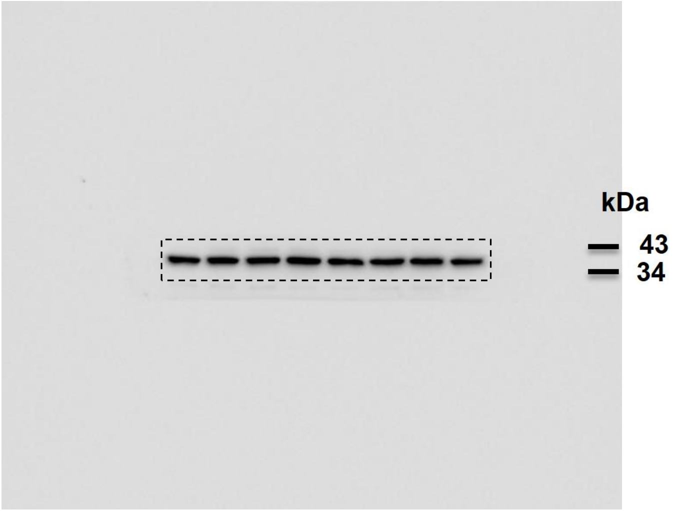

β-actin

Supplementary Figure S13. The original immunoblots of Figure 5H.

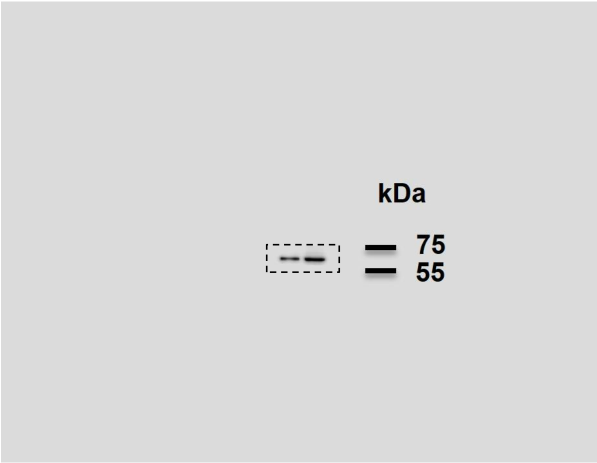

PRKACA

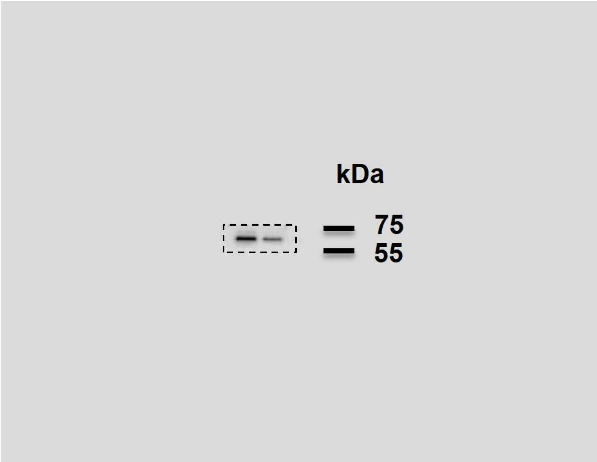

PRKACA

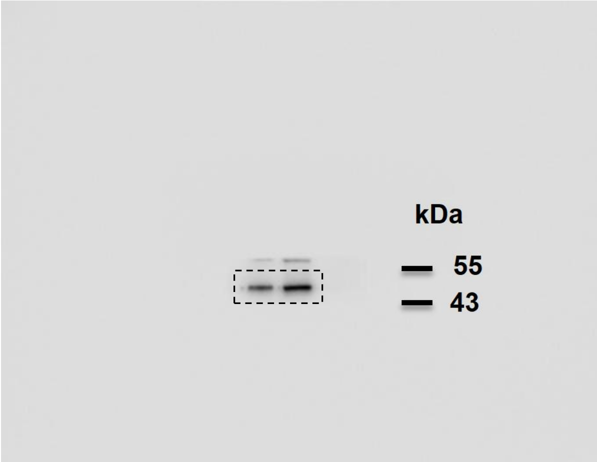

pGSK3β

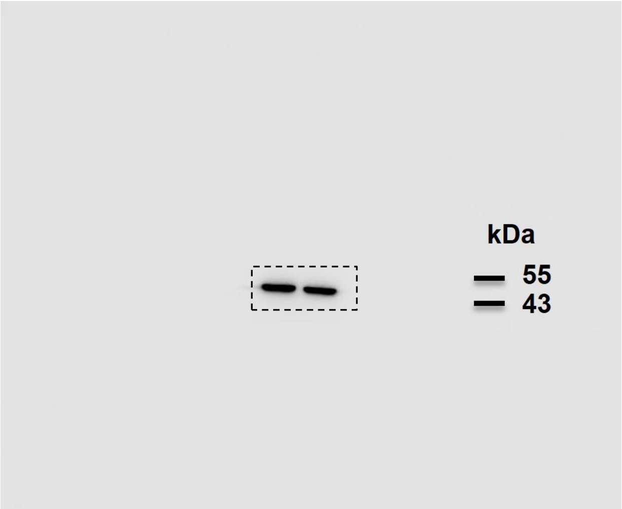

GSK3β

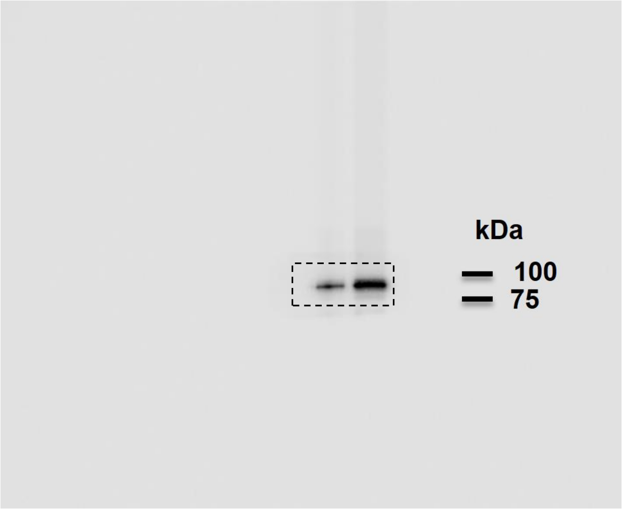

β-catenin

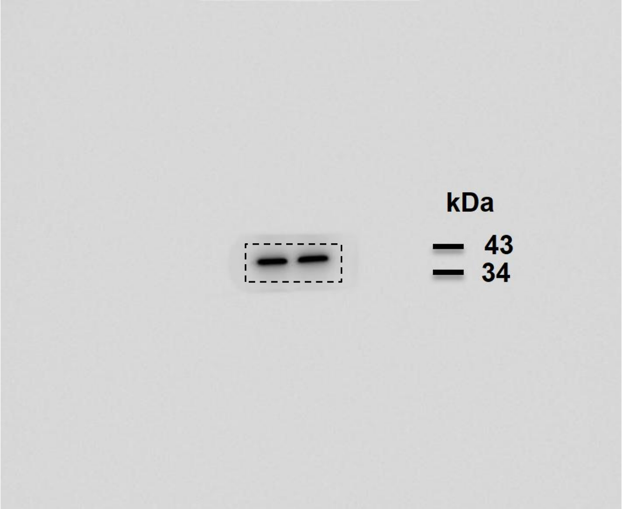

β-actin

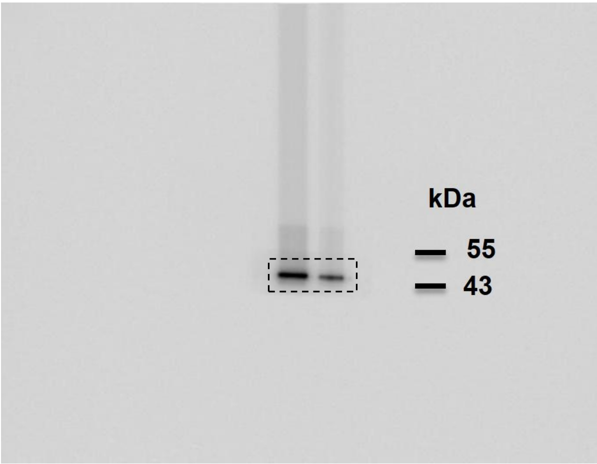

pGSK3β

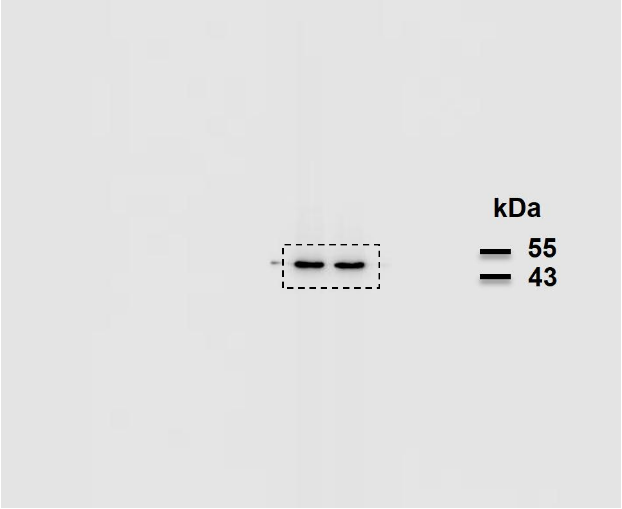

GSK3β

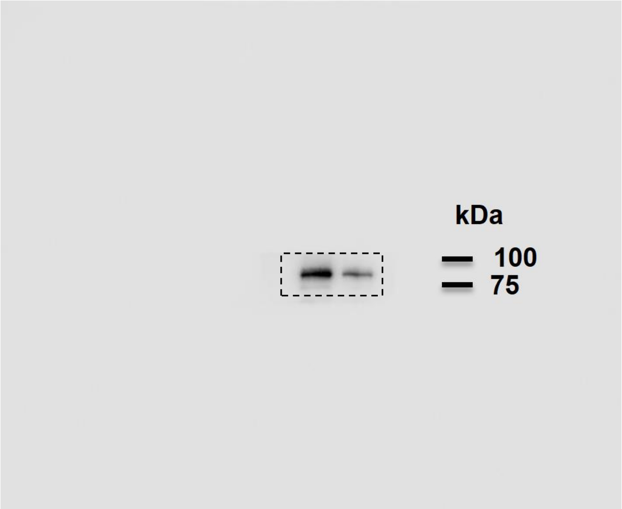

β-catenin

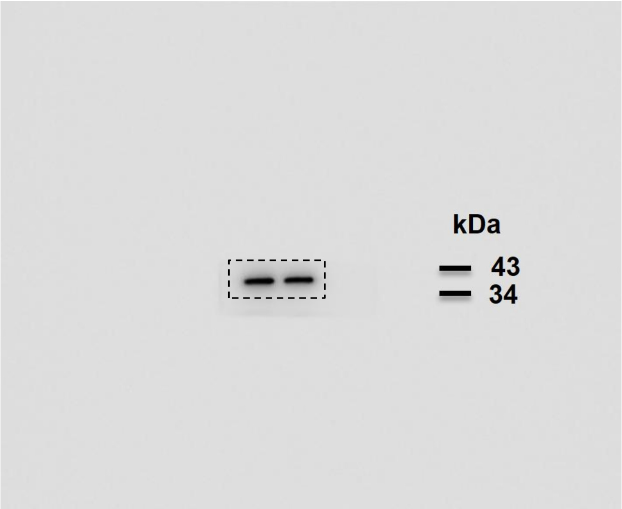

β-actin

Supplementary Figure S14. The original immunoblots of Figure 6E.

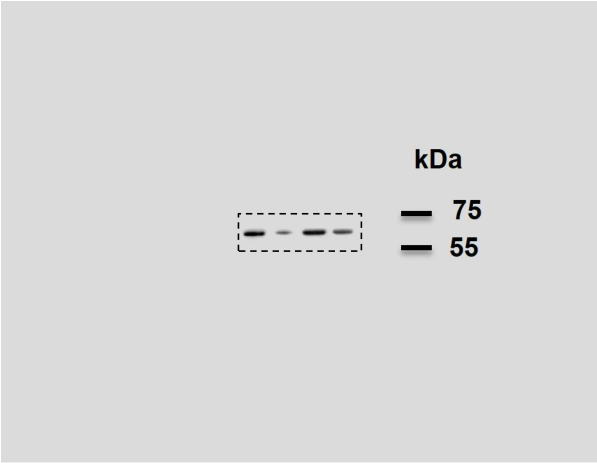

CARM1

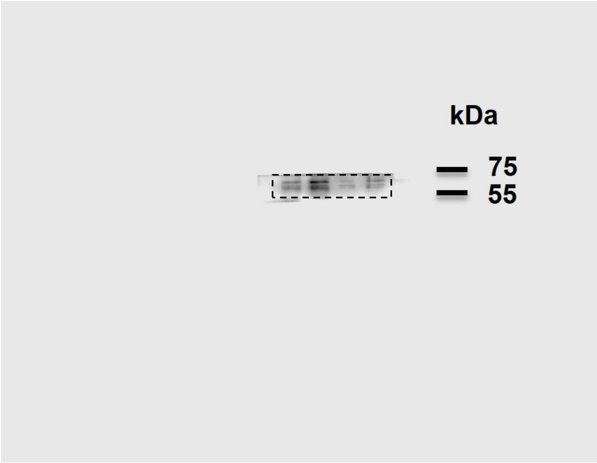

PRKACA

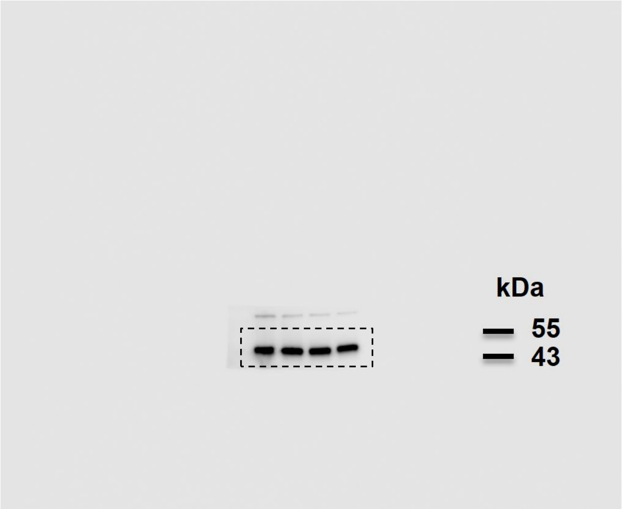

GSK3β

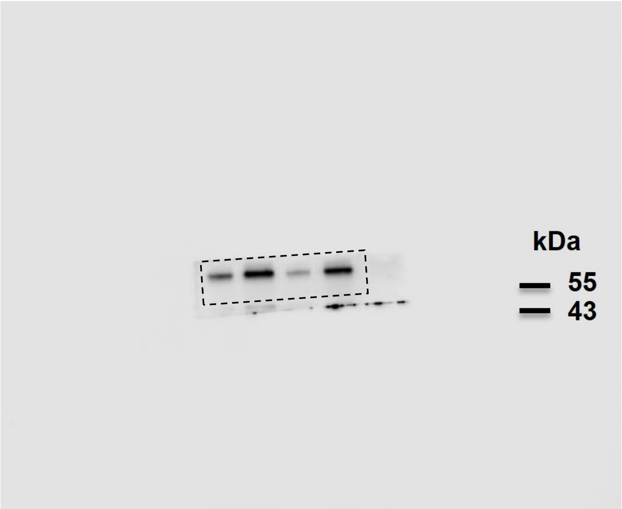

pGSK3β

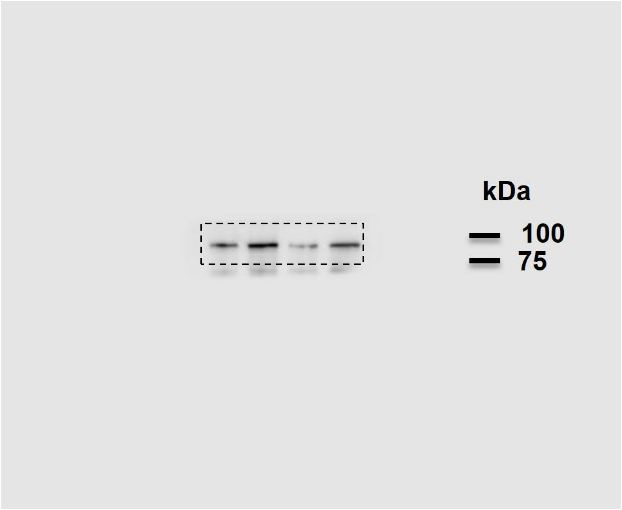

β-catenin

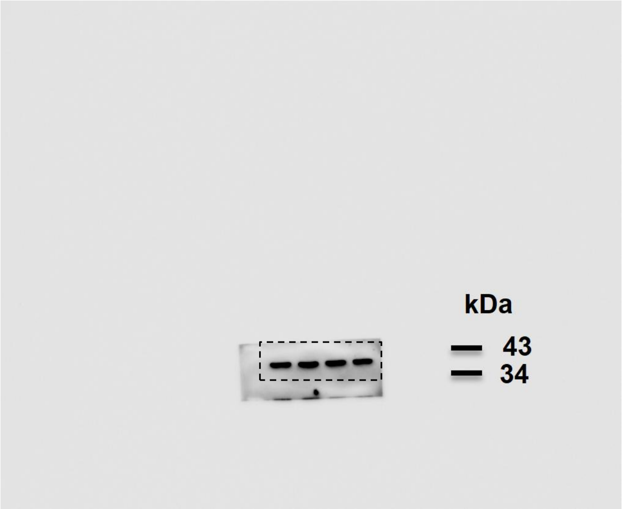

β-actin

Supplementary Figure S15. The original immunoblots of Figure 6G.

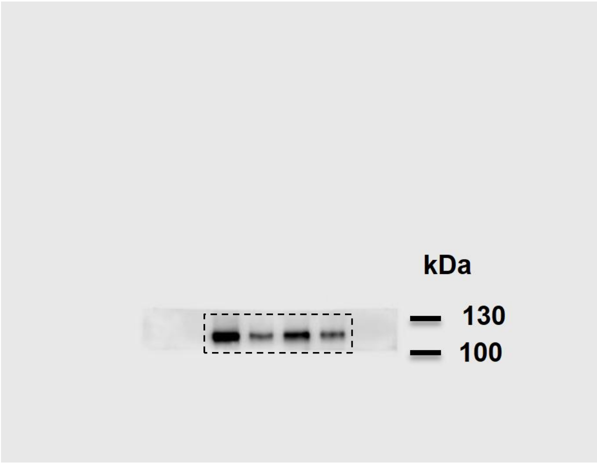

RBM5

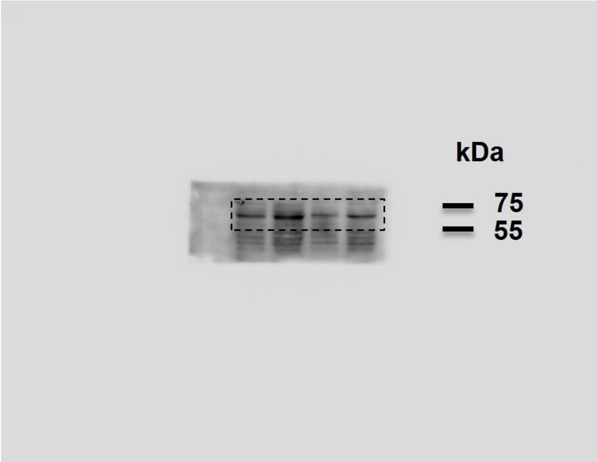

PRKACA

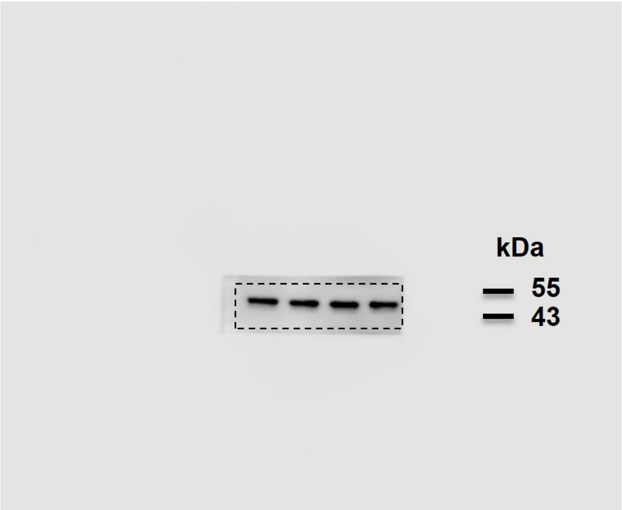

GSK3β

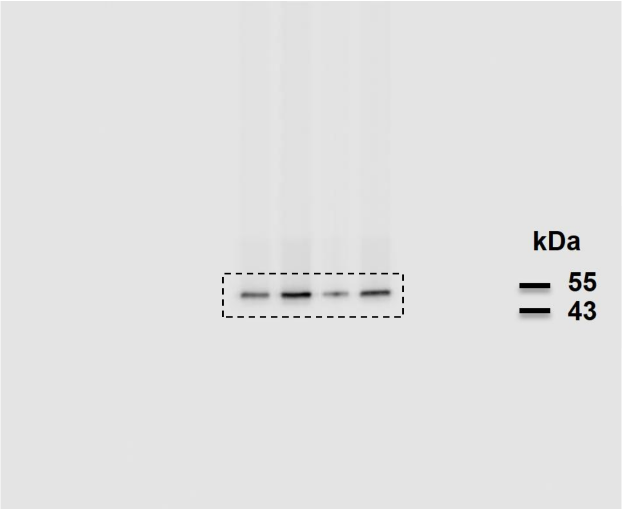

pGSK3β

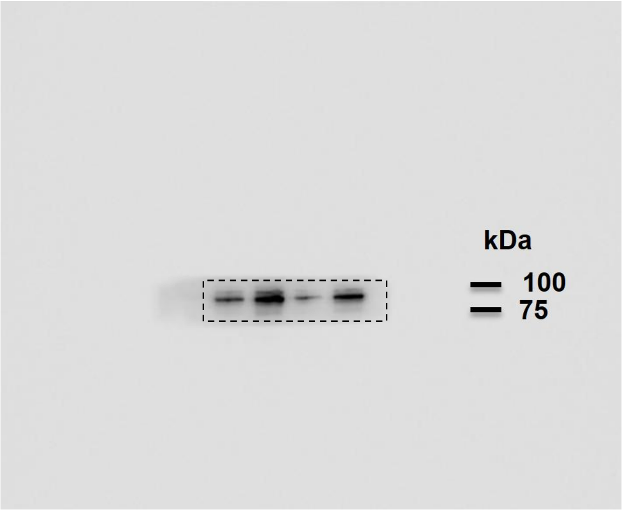

β-catenin

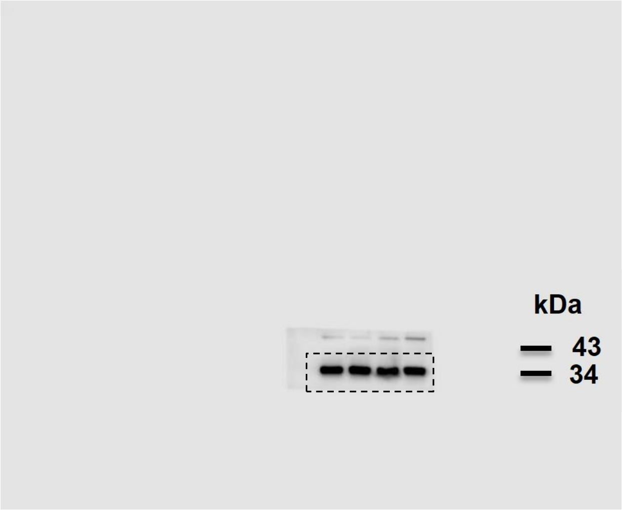

β-actin

Supplementary Figure S16. The original immunoblots of Figure 6I.

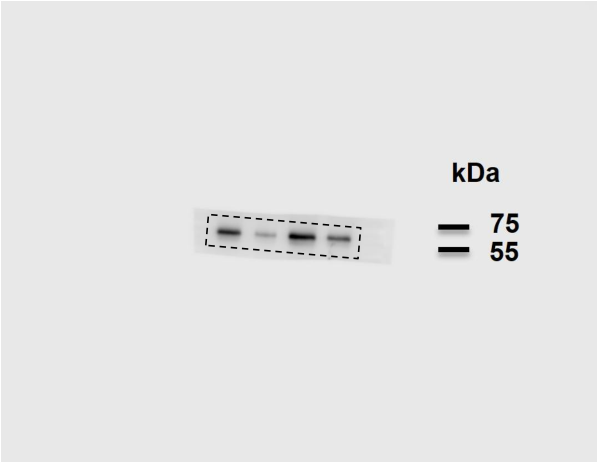

PRKACA

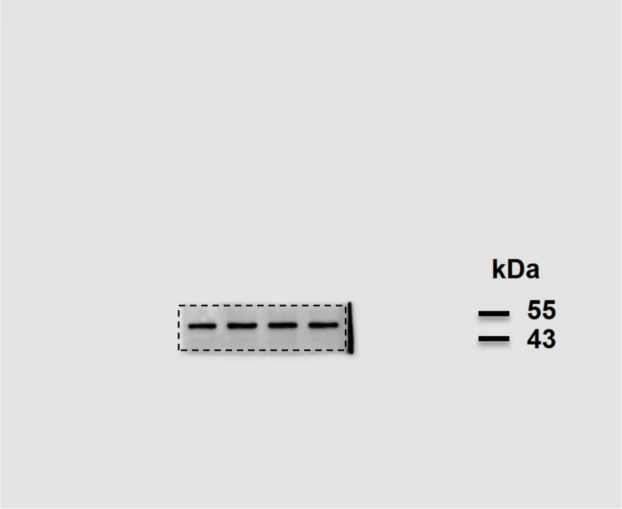

GSK3β

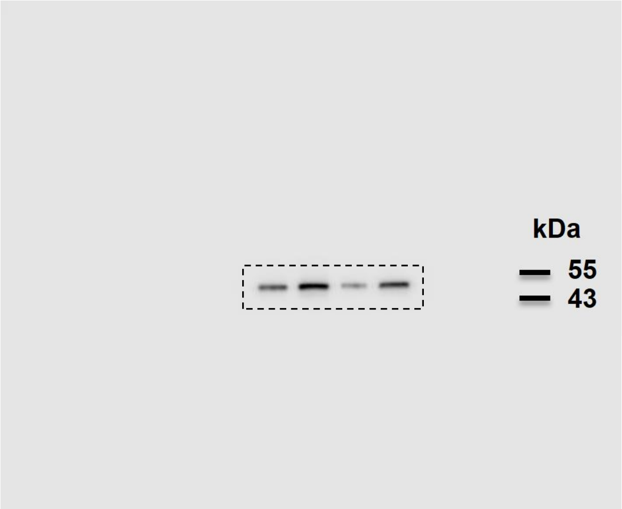

pGSK3β

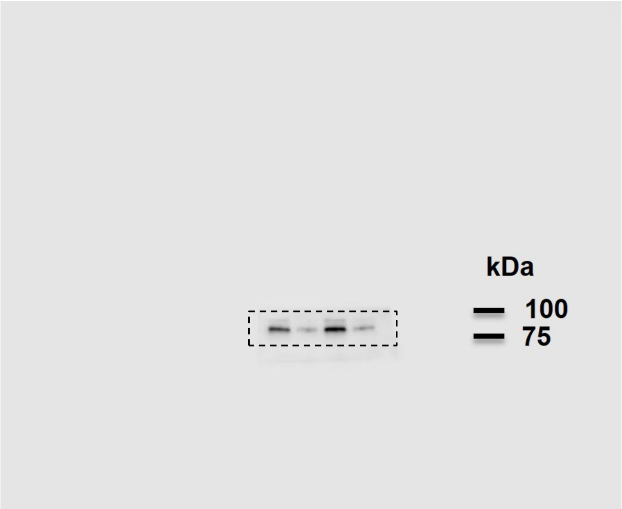

β-catenin

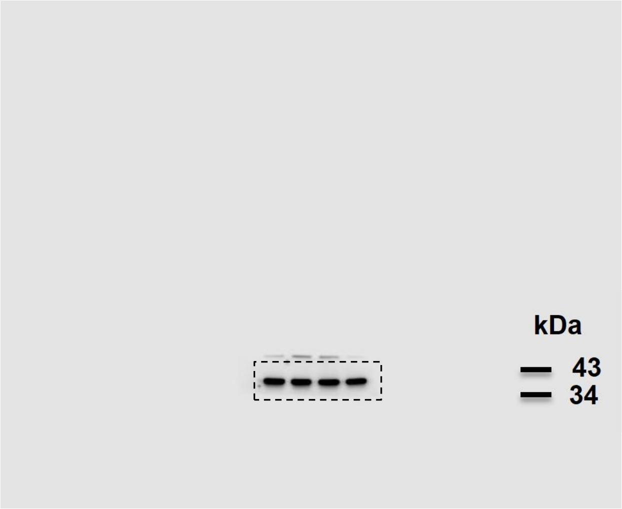

β-actin

Supplementary Figure S17. The original immunoblots of Figure 6K.

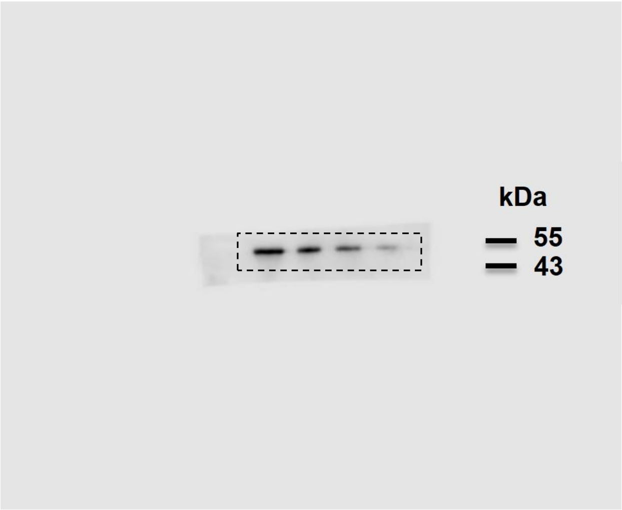

pGSK3β

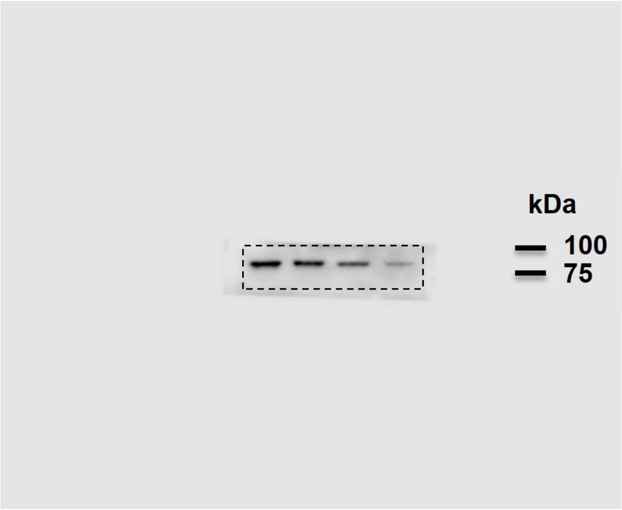

β -catenin

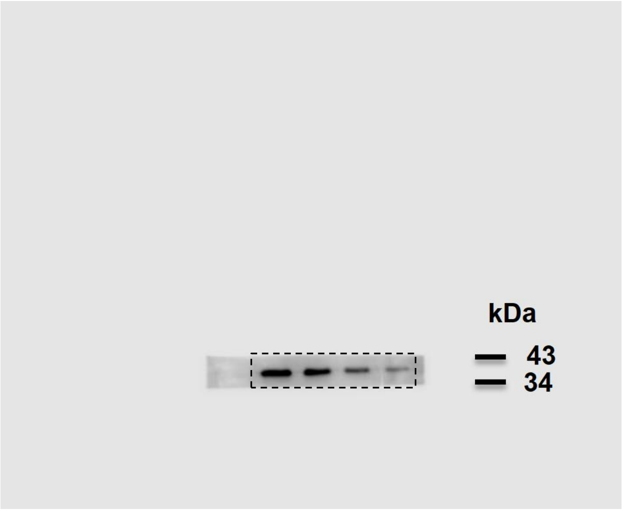

CDK4

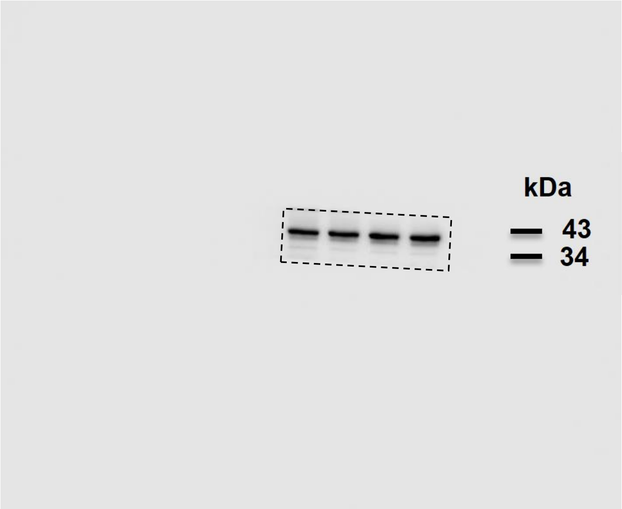

β-actin

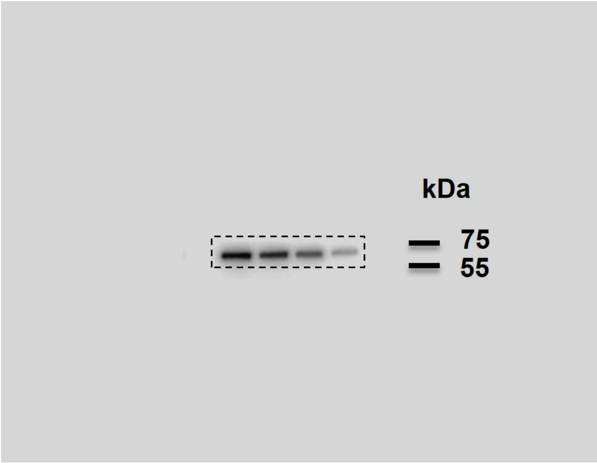

PRKACA

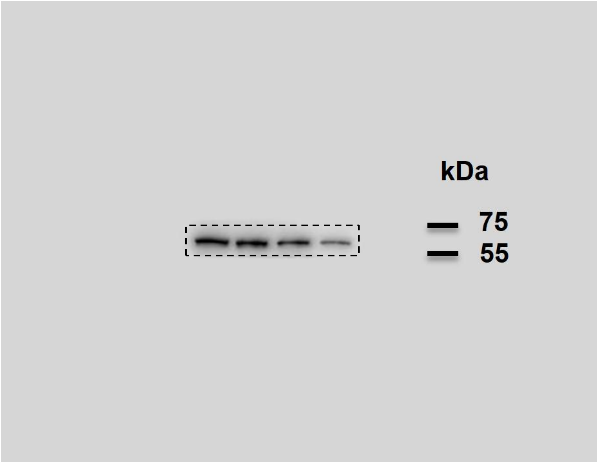

CARM1

Supplementary Figure S18. The original immunoblots of Figure 7E.
