# Supplementary material for: Downregulated RBM5 Enhances CARM1 Expression and Activates the PRKACA/GSK3β Signaling Pathway through Alternative Splicing-Coupled Nonsense-Mediated Decay
Source: Cancers (Basel). 2023 Dec 27;16(1):139. doi: 10.3390/cancers16010139 (PMC10778212; doi:10.3390/cancers16010139)
Supplement: Supplementary file 1 [file cancers-16-00139-s001.zip › Simple Summary.pdf]

Previous studies demonstrated that downregulated RBM5 promotes the progression of Bladder Cancer. Alternative splicing (AS) plays a crucial role in the progression of cancer by promoting nonsense-mediated mRNA decay (NMD). However, whether RBM5 modulates the progression of BC through AS-NMD remains unexplored. The present study revealed that RBM5 negatively regulates the expression of CARM1 by binding directly to its mRNA and participating in the NMD process of CARM1 mRNA in BC cells. CARM1 mediates the activation of Wnt/β-catenin and RBM5 by promoting the phosphorylation of GSK3β. Protein kinase catalytic subunit alpha (PRKACA) acts as a phosphorylated kinase of GSK3β and is regulated by CARM1 at the transcription level. The results proved that a regulatory mechanism for Wnt/β-catenin activation through the RBM5/CARM1/PRKACA axis and identified a new potential target for treating BC.
