# Supplementary material for: Downregulated RBM5 Enhances CARM1 Expression and Activates the PRKACA/GSK3β Signaling Pathway through Alternative Splicing-Coupled Nonsense-Mediated Decay
Source: Cancers (Basel). 2023 Dec 27;16(1):139. doi: 10.3390/cancers16010139 (PMC10778212; doi:10.3390/cancers16010139)
Supplement: Supplementary file 1 [file cancers-16-00139-s001.zip › Supplementary material.pdf]

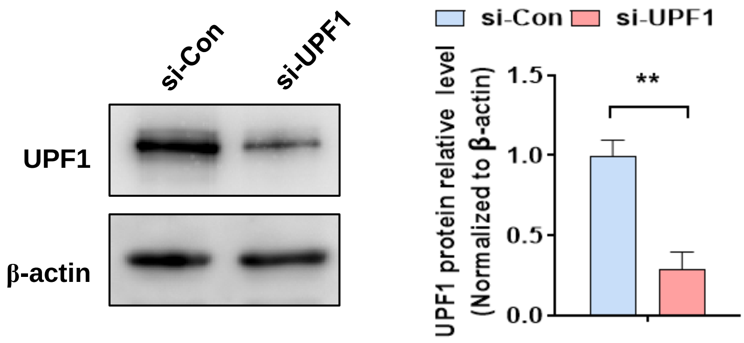

Supplementary Figure S1 T24 cell transfected with si-UPF1 or si-Con and Western blot was used to examine UPF1 protein expression.

Supplementary Table 1 Primers used in this study.

Name	Sequences
TBC1D3G-F:	TACGAAAAGGGACACCGAGC
TBC1D3G-R:	TCTCCCAGCATATCCACCCA
RADIL-F:	ACAGCCTGGTGTATGTGCTC
RADIL-R:	CCCACCTCGGAGAAGTTGAC
KCNJ18-F:	CAGTCAGCTTTCCGTCCTGT
KCNJ18-R:	AGTAGTGGCTGCCATTTCCC
LLPHP3-F:	GCGTGATCCTGAAGTGTGTG
LLPHP3-R:	TGGCAATGTTTGGGTTTGGG
KISS1R-F:	TCAGCATCTGGGTAGGCTCT
KISS1R-R:	CCAGCACCTGCCCCTG
MANSC4-F:	AAGCATGGGGTGGACATCAG
MANSC4-R:	AGGATGGAGCCTCTGTGGAT
PROX2-F:	CACATCCTACAGGCTGCCAA
PROX2-R:	TGCAGCGGTTGAACTTTTGT
CARM1-F:	ATCGCCCTCTACAGCCATGA
CARM1-R:	ACTGGATGAGGACGCTGTTG
NLRP14-F:	ACTCTCAGGCTGGAATCTTGC
NLRP14-R:	AGCTTCTGGGCTTCCTCATCA
FAM124A-F:	CTCCACGAGCAGTGAGCTTT
FAM124A-R:	GTCGTAGCGACAGTAGACGG
GAPDH-F:	TGCACCACCAACTGCTTAGC
GAPDH-R:	GGCATGGACTGTGGTCATGAG
PRKACA-prom-F:	GTCACGCGTgtctccgggccagggtcagggaccc
PRKACA-prom-R:	GCA/AGCTTgacgcccccgcagcccgccg
CARM1-CDS-F:	GGCAGCCTGTGGTGGACACATTTG
CARM1-CDS-R:	ATGGAGCCGATGAAAGCAACGTC
CARM1-E9M-F:	GCCTGTGGTGGATAGAAATCC
CARM1-E9M-R:	ATGGAGCCGATGAAAGCAACG
T7-F	TAATACGACTCACTATAGGG
BGH-R	TAGAAGGCACAGTCGAGG
pcDNA3.1-R	TCCACTAGTCCAGTGTGGTG
V5-R	GAGGGTTAGGGATAGGCTTAC
Si-Con-F:	UUCUCCGAACGUGUCACGUTT
Si-Con-R:	ACGUGACACGUUCGGAGAATT
Si-UPF1-F:	GCGUCAUCAUUGUGGGCAATT
Si-UPF1-R:	UUGCCCACAAUGAUGACGCTT
